# Response to a novel type II RAF inhibitor in diffuse leptomeningeal glioneuronal tumor with BRAF fusion

**DOI:** 10.1093/oncolo/oyaf093

**Published:** 2025-05-16

**Authors:** Brie M Chun, Sandra Youngworth, Vicki Abtin, David Stull, Jay Starkey, Shivaani Kummar

**Affiliations:** Knight Cancer Institute, Oregon Health & Science University, Portland, OR 97239, United States; Knight Cancer Institute, Oregon Health & Science University, Portland, OR 97239, United States; Knight Cancer Institute, Oregon Health & Science University, Portland, OR 97239, United States; Knight Cancer Institute, Oregon Health & Science University, Portland, OR 97239, United States; Diagnostic Radiology, Oregon Health & Science University, Portland, OR 97239, United States; Knight Cancer Institute, Oregon Health & Science University, Portland, OR 97239, United States

## Abstract

**Background:**

Diffuse leptomeningeal glioneuronal tumor (DL-GNT) is a rare disease which is more often diagnosed in children and adolescents than adults. Activation of the MAPK/ERK pathway is implicated in the majority of cases, and BRAF fusions are the most common genetic alteration. BRAF fusions result in dimerization and constitutive downstream MAPK/ERK activity, against which type I RAF inhibitors have limited efficacy. Type II RAF inhibitors stabilize RAF in an inactive conformation and inhibit both dimer protomers, thus inhibiting downstream MAPK/ERK activity in the setting of BRAF fusions.

**Case Presentation:**

A previously-healthy 33 year old man was diagnosed with DL-GNT, which harbored a pathogenic *BRAF:KIAA1549* gene fusion. He was initially treated with a MEK inhibitor but developed drug-related cardiotoxicity. Without treatment, he developed significant functional limitations due to leptomeningeal disease. A compassionate use indication was pursued for an investigational CNS-penetrant type II BRAF inhibitor, tovorafenib. Within 3 months of initiating the medication, the patient experienced notable gains in functional status and with over 12 months of treatment has been able to rejoin recreational activities.

**Conclusions:**

This case highlights the importance of tumor molecular characterization, particularly in rare tumors, whereby identification of the *BRAF:KIAA1594* gene fusion led to an appropriate selection of a type II BRAF inhibitor.

**Key Points**

- Diffuse leptomeningeal glioneuronal tumor (DL-GNT) is a rare tumor more commonly presenting in children and adolescents, but is characterized by BRAF fusions, most commonly *BRAF:KIAA1549*
- Type II but not type I BRAF inhibitors are able to silence oncogenic BRAF dimer signaling which can arise with activating BRAF fusions
- Tovorafenib is a brain-penetrant type II BRAF inhibitor in development for adult solid tumors

## Patient Story

**Figure 1.**
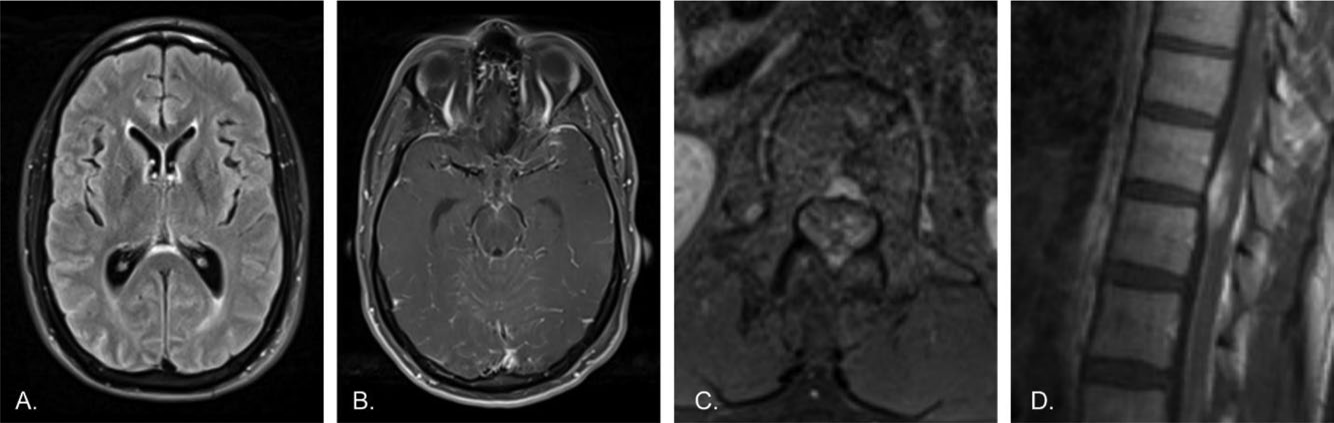
A previously healthy 33-year-old male presents with new-onset encephalopathy, headaches, and seizures. A) Axial T2-FLAIR image of the brain shows slight periventricular and sulcal hyperintensity. B) Axial post-contrast T1 image of the brain shows diffuse subtle leptomeningeal enhancement, including along the midbrain surface and cerebellar folia. There was no associated diffusion restriction (not shown). C) Axial post-contrast T1 image of the lumbar spine shows enhancement of the cauda equina and about the conus. D) Sagittal post-contrast T1 image of the lower thoracic spine shows diffuse enhancement of the spinal cord surface with more mass-like enhancement involving the ventral cord.

A previously healthy 33-year-old male presented with new-onset encephalopathy, headaches, nausea, vomiting, bilateral cranial nerve dysfunction with facial palsy. MRI studies were notable for non-specific subtle hyperintensities in the subarachnoid spaces of the frontal, parietal, occipital, and temporal lobes; and diffuse leptomeningeal enhancement along the conus medullaris and cauda equina nerve roots (Figure 1). Multiple sub-specialists were involved in the diagnostic evaluation, and the differential diagnosis included infection, malignancy, and sarcoidosis but was elusive despite extensive serological testing and repeated lumbar punctures for spinal fluid analysis. He was treated empirically with antivirals, antibiotics, and steroids without meaningful improvement and required a lumbar drain to manage a communicating hydrocephalus. He developed status epilepticus, and underwent a craniotomy for meningeal biopsy, which was non-diagnostic. Nearly two months after his initial presentation, with persistent severe symptoms, a T10-11 laminectomy was performed to biopsy a suspicious intradural lesion. Pathologic interpretation and confirmatory second opinion led to a diagnosis of diffuse leptomeningeal glioneuronal tumor (DL-GNT).

**Figure 2.**
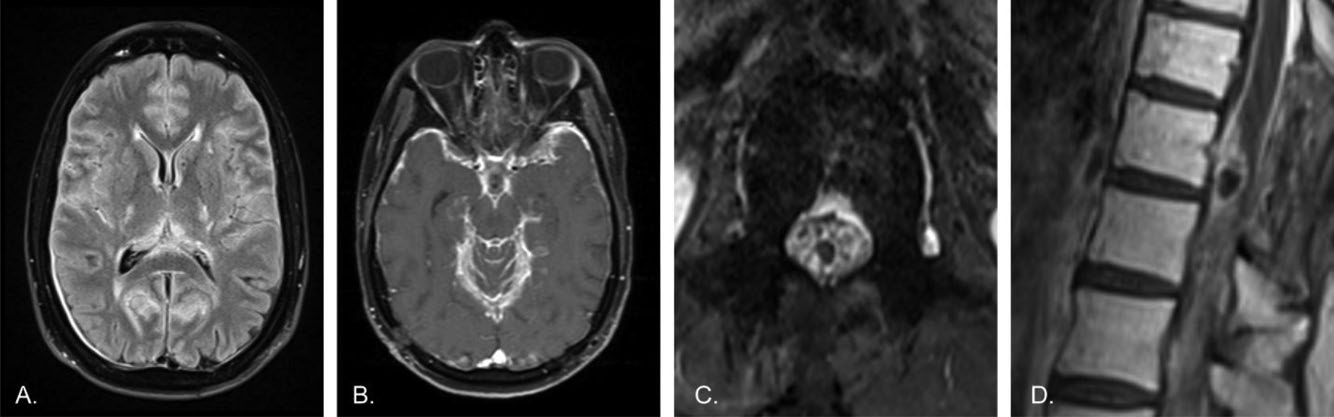
Worsening disease following radiation therapy and cessation of the MEK inhibitor trametinib due to side effects. A) Axial T2-FLAIR image of the brain shows worsened leptomeningeal hyperintensity. Dural thickening over the right occipital lobe is likely related to a craniotomy (not shown). B) Axial post-contrast T1 image of the brain demonstrates much thicker diffuse leptomeningeal enhancement. C) Axial post-contrast T1 image of the lumbar spine shows thick, diffuse enhancement of the cauda equina, now clearly outlining the conus as a dark circle in the center of the canal. D) Sagittal post-contrast T1 image of the lower thoracic spine demonstrates much thicker enhancement of the cord surface, and continued enhancement of the dorsal mass, which also shows biopsy changes. Note the medullary hyperintensity, consistent with history of radiation therapy.

The patient initially received definitive radiation to the brain and spine, but eventually developed disease progression within the thoracic spinal cord and at the lumbosacral nerve roots. To target the BRAF signaling pathway, which is implicated in the pathogenesis of DL-GNT, he was treated with the MEK inhibitor trametinib with initial disease stabilization. He developed a ventriculoperitoneal shunt infection as well as drug-induced cardiomyopathy which necessitated trametinib discontinuation and prohibited re-challenge despite eventual improvement in cardiomyopathy. Without treatment, the patient's disease progressed, and he developed worsening symptoms and a decline in functional status (Figure 2). His cognitive function declined, he developed diplopia, and gait instability leading to loss of balance when turning. He unintentionally lost significant weight, required standby assistance or four-wheel walker to ambulate safely for short distances, and wheelchair for longer distances.

## Molecular Tumor Board

### Molecular testing results and interpretation

As part of this tumor’s pathologic evaluation, the specimen was submitted for molecular analysis using an institutional next-generation sequencing panel (University of California, San Francisco, CA). The tumor harbored a pathogenic *ATRX* pI1049fs mutation, *BRAF:KIAA1549* gene fusion, monosomy of chromosome 1p and distal 19q, and trisomy of chromosome 1q. Its tumor mutation burden: 5.5 mut/Mb.

Diffuse leptomeningeal glioneuronal tumor (DL-GNT) is a rare disease, specified recently within the 2016 WHO classification of tumors of the central nervous system (CNS).^1^ The true incidence is unknown due to a limited number of cases. It is more frequently diagnosed in children and adolescents, with a median age of diagnosis between 5 and 11 years^2,3^ though it can rarely occur in adults.^4-6^ DL-GNT typically presents with diffuse leptomeningeal disease and variable degrees of parenchymal involvement. Clinical manifestations of leptomeningeal disease are often multifocal and commonly involve headache related to increased intracranial pressure due to hydrocephalus or meningeal irritation, altered mental status, cranial neuropathies, cerebellar dysfunction and potentially seizure. Most frequently the tumor displays relatively slow growth rates and histologically appears as a low-moderate cellularity tumor with low mitotic activity,^1,7^ though a subset appears to develop aggressive features^8^ and clinical manifestations can be highly morbid regardless of the tempo of disease progression or disease burden.

For up to 80% of DL-GNT cases, activation of the MAPK/ERK signaling pathway is implicated in tumorigenesis, with the most common genetic alteration being a *BRAF:KIAA1549* fusion.^7,9^ A gain of chromosome 1q is an adverse prognosticator for progression-free and overall survival in DL-GNT.^10^ Monosomy of 1p often co-occurs with *BRAF:KIAA1549* fusions.^9^

### Functional and clinical significance of *BRAF:KIAA1549* fusion

The *BRAF:KIAA1549* fusion results in the loss of a regulatory domain and leads to RAS-independent activity and thus activation of downstream MEK/ERK functions.^11,12^ This fusion is also found in pilocytic astrocytoma,^13^ and high-grade astrocytomas with piloid features.^14^ The discovery of BRAF activating fusions in pediatric-type tumors of the CNS has led to interest in therapeutically targeting the MAPK/ERK pathway.^7,12,15-18^ Although FDA-approved type I RAF inhibitors are effective against certain BRAF alterations, such as V600E which displays RAS-independent monomer signaling activity, they are not effective against BRAF fusions that result in RAS-independent dimer signaling.^11,18^ Type I RAF inhibitors may also lead to paradoxical downstream activation.^19^ As was the case for this patient, targeting the pathway using a MEK inhibitor would theoretically be effective, as this protein is downstream of the BRAF fusion. Unfortunately, drug-related toxicities limited the duration of treatment with trametinib.

Type II RAF inhibitors are able to silence constitutive downstream MAPK/ERK activity resulting from BRAF dimerization.^11,18^ Tovorafenib is a selective, CNS-penetrant type II RAF inhibitor dosed once-weekly and is available in tablet and oral suspension formulations. It is being evaluated in an ongoing phase 2 open-label trial (FIREFLY-1 [NCT04775485]) in RAF-altered pediatric low-grade gliomas (pLGG) and advanced solid tumors. Preliminary results from the 77 patients in the registrational cohort (June 5, 2023 data cutoff) demonstrated a 51% overall response rate (as assessed by Response Assessment in Pediatric Neuro-Oncology Low-Grade Glioma (RAPNO) criteria); median duration of response was 16.6 months; and median time to response was 3.0 months.^20^ Tovorafenib received accelerated approval in April 2024 by the US FDA in patients 6 years of age and older with relapsed and refractory pLGG. It has also been explored as a monotherapy or in combination with other agents for use in other pediatric and adult solid tumors.^21,22^

## Patient update

**Figure 3.**
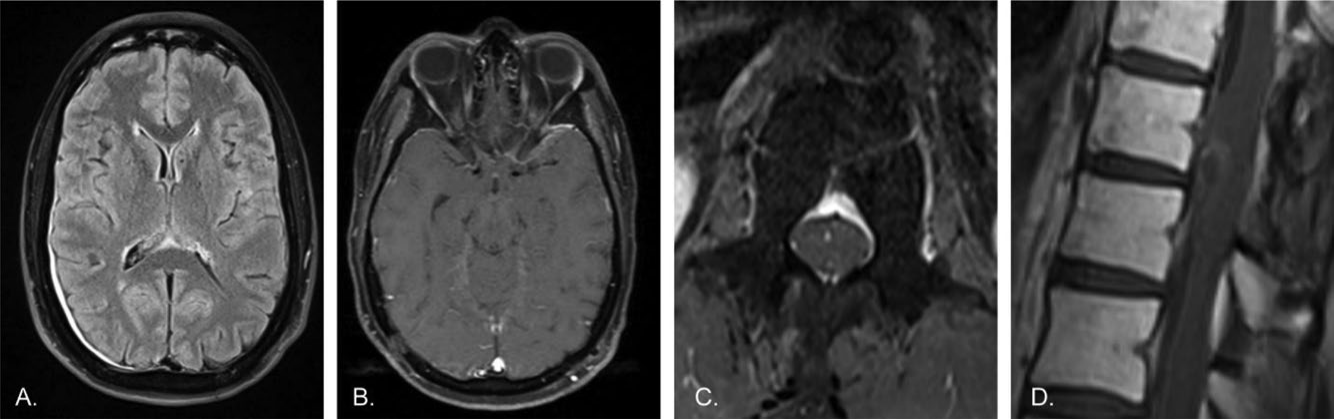
Restaging MRI 3 months following initiation of C1D1 treatment with tovorafenib. A) Axial T2-FLAIR image of the brain shows persistent but markedly reduced leptomeningeal hyperintensity compared to prior. Persistent dural thickening is again likely post-surgical in nature. B) Axial post-contrast T1 image of the brain demonstrates markedly decreased leptomeningeal enhancement, with no clear residual enhancement beyond what may represent normal background. C) Axial post-contrast T1 image of the lumbar spine shows no discernible enhancement of the cauda equina, now appearing essentially normal. D) Sagittal post-contrast T1 image of the lower thoracic spine shows a significant reduction in cord surface enhancement, with only faint residual enhancement of the previously noted mass-like lesion along the ventral cord. Overall, findings are significantly improved compared to all previous time-points, including the initial presentation.

The patient sought enrollment onto a phase I clinical trial with tovorafenib (FIRELIGHT-1 [NCT04985604]) which proffered a compelling rationale for treatment based on the drug’s ability to penetrate the blood-brain barrier and the patient’s *BRAF:KIAA1549* fusion. Unfortunately, the patient did not meet eligibility criteria to enroll due to his previous treatment with trametinib and his performance status. A compassionate use protocol for the investigational agent was opened for the patient to begin treatment. Gradually his symptoms began to improve, with notable functional gains within the first 3 months of treatment. With over 12 months of treatment, he is now able to ambulate unassisted, join physical exercise classes, enjoy recreational hikes, and perform light housework. Restaging MRI studies of the brain and spine have since demonstrated disease improvement and stabilization without new evidence of disease (Figure 3).

## Discussion

This case illustrates how molecular-targeted therapies can have a striking impact on a patient’s disease course. DL-GNT is an exceedingly rare tumor without established standard systemic treatments guided by prospective clinical trials. Due to its rarity, there have not been clinical trials to establish a standard of care. Conventional treatment options include chemotherapy, radiation, and supportive measures.^23-25^ A recent review described median overall survival among 63 published cases as just 19 months, and moreover an age at diagnosis greater than 9 years to be independently associated with shorter overall survival.^2^ Although the diagnosis can be elusive due to challenges obtaining diagnostic tissue, the tumors are characterized by alterations in BRAF signaling, commonly with a *BRAF:KIAA1549* gene fusion. Oncogenic signaling by RAS-independent RAF dimers is inhibited by type II RAF inhibitors, and tovorafenib represents a selective CNS-penetrant agent. The development of effective treatments against BRAF dimers makes establishing the diagnosis and obtaining molecular characterization paramount. Though this patient was treated in a compassionate use setting, we hope that this experience will add to a growing body of knowledge that type II RAF inhibition can be effective for patients with DL-GNT.

## Data Availability

The data underlying this article cannot be shared publicly due to concerns regarding the privacy of individuals that participated in the study. The data will be shared on reasonable request to the corresponding author.
